# “So Much Comes Up”: Emotion Regulation in Psychotherapy Addressing Existential, Spiritual and Religious Themes

**DOI:** 10.3390/bs16050685

**Published:** 2026-04-30

**Authors:** Joke C. van Nieuw Amerongen, Carolien van Stam, Anne-Mieke Romkes-Bart, Arjan W. Braam, Hanneke Schaap-Jonker, Bart van den Brink

**Affiliations:** 1 School of Religion and Theology, Faculty of Social Sciences and Humanities, Vrije Universiteit Amsterdam, 1081 HV Amsterdam, The Netherlands; h.schaap@kicg.nl; 2 Center for Research and Innovation in Christian Mental Health Care, 3871 MR Hoevelaken, The Netherlands; 3 Eleos Mental Health Care, 3871 MR Hoevelaken, The Netherlands; carolien.vanstam@eleos.nl (C.v.S.);; 4 Dimence Mental Health Care, 8043 RR Zwolle, The Netherlands; 5 Department of Acute Psychiatry, Altrecht Mental Health Care, 3512 PG Utrecht, The Netherlands; a.braam@altrecht.nl; 6 Department of Humanist Chaplaincy Studies, University of Humanistic Studies, 3512 HD Utrecht, The Netherlands; 7 GGz Centraal, 3818 EW Amersfoort, The Netherlands; b.vandenbrink@ggzcentraal.nl; 8 Department of Practical Theology, Theological University Apeldoorn, 7316 BT Apeldoorn, The Netherlands; 9 University Medical Center Utrecht, 3584 CX Utrecht, The Netherlands

**Keywords:** meaning in life, spirituality, religion, psychotherapy, emotion-regulation, SPIRIT, CBT

## Abstract

Existential, spiritual, and religious themes often evoke strong emotions in therapy, yet little is known about how clients’ emotion regulation relates to these aspects. Spiritual psychotherapy for inpatient residential and intensive treatment (SPIRIT) integrates meaning in life within a cognitive-behavioral treatment (CBT) framework in acute and intensive mental health care and provides an appropriate context for examining this. This qualitative study explores: (1) clients’ beliefs about expressing, managing, or suppressing emotions related to meaning in life, spirituality, or religion (MSR); (2) how emotion regulation strategies (e.g., reappraisal, acceptance, and distress tolerance) are influenced by addressing MSR in therapy; and (3) whether engaging with MSR activates emotion regulation mechanisms for clients’ experienced distress. We analyzed 118 client evaluation forms and 19 semi-structured client interviews using a thematic approach informed by emotion regulation theory. SPIRIT-CBT made implicit beliefs about (MSR-related) emotion regulation explicit, and group interactions sometimes led to changes. Clients showed various regulation strategies, for example: MSR-based reappraisal, connectedness, reflection, and positive refocusing. However, emotional tension and suppression were also reported. Particularly from the interviews, it emerged that the therapy facilitated regulation mechanisms, including narrative processing, perspective shifting, sense-making, and social belonging. Focusing on MSR and existential themes addresses an important gap in mental health care and may contribute to supporting clients’ emotional recovery and overall well-being.

## 1. Introduction

Meaning in life is a broad construct encompassing understanding or coherence, purpose, and significance (George & Park, 2016; Steger, 2022). Schnell et al. (2019), a prominent European researcher in existential psychology, proposes adding “belonging” as a key element to the other three. For many individuals, in the context of mental health, relationships are closely tied to experiences of meaning in life (Glaw et al., 2017; Malycha & Krok, 2024). Relationships belong to what people consider most important in their lives, which is often intertwined with spirituality and religion (Krok, 2015; O’Sullivan et al., 2024; Krok et al., 2025). Religion has been conceptualized as the institutional and culturally determined expression of spirituality, while spirituality can be defined as the individual search for the sacred (Pargament, 1997, 2013; Pargament & Pomerleau, 2025). Although recent studies rarely attempt to formulate more comprehensive definitions of religion and spirituality, both empirical and review-based research indicate that these constructs are conceptualized in diverse ways and lack a shared definition (Paul Victor & Treschuck, 2020; Chagas et al., 2023). A recent study on spiritual health in Canada highlights that, while the literature on spirituality and health has grown over the past decades, it remains underdeveloped in terms of conceptualization, operationalization, and integration with broader health frameworks. This gap points to the need for greater conceptual clarity, attention to cultural perspectives, and a nuanced understanding of the ways in which spirituality and religion are understood (Boutros et al., 2026). However, it is clear that meaning in life is highly intertwined with the fields of religion and spirituality (Krok, 2015; Small, 2023), which supports the inclusion of the topic in the conceptual lens of MSR for the current study. Emotions are closely involved in this area (Tang et al., 2013; Rybarski et al., 2023). In the current study, we use the term MSR to encounter meaning in life, spirituality, and religiosity.

Within the framework of MSR, understanding, coherence, significance, and belonging acquire additional psychological weight. They are not only cognitive or motivational constructs, but also existential anchors that guide interpretation of suffering, inform coping strategies, and shape emotional experiences (Pargament & Pomerleau, 2025). Skrzypińska (2022) integrates psychological theory and research to conceptualize these aspects, proposing two models that link personal beliefs and meaning systems to cognitive and personality processes. These models view MSR as multi-factorial, interacting with cognitive processes and personality, and having behavioral consequences regarding attitudes toward the sacred or non-sacred aspects of existence. These perspectives are directly relevant for understanding how meaning, spirituality, and religiosity function as emotion regulation mechanisms in therapeutic contexts.

In the context of mental illness, individuals encounter both support and struggles related to MSR (Exline et al., 2000; Krok et al., 2024). These so-called ‘religious and spiritual struggles’ often are felt as core emotional experiences (van Nieuw Amerongen-Meeuse et al., 2022). Sometimes these experiences are congruent with clients’ psychological distress, while at other times they can be in tension or even controversial or compensating. Religious/spiritual (R/S) struggles influence well-being (Pargament et al., 2025), with religious support and meaning-making acting as mediators: when support is felt, well-being improves, but when support is lacking, well-being declines (Krok et al., 2024; Zarzycka et al., 2020). R/S struggles are emotionally challenging and, regardless of religiousness, are linked to increased and diverse coping efforts (Wilt et al., 2024). One study showed that clients suffering from major depression who experienced R/S struggles reported persistent high levels of R/S struggles after CBT, indicating that specific attention towards these struggles in therapy might be needed (Kéri, 2025).

The importance of attending MSR in therapy has increasingly been recognized (Główczyński et al., 2026; Milner et al., 2019; Pargament et al., 2005; Vieten et al., 2023). In recent decades, spiritually integrated therapies have emerged as approaches that explicitly incorporate MSR into psychotherapy (Pargament, 2013; Richards et al., 2023). Studies show that these therapies are more, or at least equally, effective than standard approaches in reducing psychological distress, and that they result in greater spiritual well-being (Captari et al., 2018; Bouwhuis-Van Keulen et al., 2024). Generally, spiritually integrated interventions utilize clients’ beliefs and meaning-making processes as a resource to cope with distress, regulate emotions, and find coherence in challenging experiences. Spiritual psychotherapy for inpatient, residential, and intensive treatment is an example of meaning-integrated psychotherapy, using a cognitive-behavioral framework to address and affect MSR-related emotions and behavior in acute and intensive psychiatric settings (SPIRIT-CBT, C. Marmarosh et al., 2024; Rosmarin et al., 2019; Rosmarin & Kaufman, 2025).

Cognitive-behavioral therapy (CBT), since its origin, has been developed in principles of behavioral and cognitive psychology (Beck, 2011; Cambridge Guide to CBT, 2025). Recent evidence indicates that improvements in emotion regulation during CBT predict subsequent reductions in social anxiety symptoms, suggesting changes in emotion regulation are a mechanism in CBT outcomes (Garke et al., 2025). Research indicates that an important mechanism through which CBT exerts its therapeutic effects is by changing emotion regulation strategies and underlying processes (Palmieri et al., 2022; Reinholt et al., 2025). Earlier research showed that CBT reduced anxiety and improved worry regulation in youth (Suveg et al., 2009), and recent studies continue to confirm improvements in emotion regulation strategies—specifically decreases in expressive suppression and increases in cognitive reappraisal—significantly associated with reductions in anxiety (Knowles & Tolin, 2024). Furthermore, earlier research demonstrated that CBT could increase cognitive reappraisal in clinically depressed clients (Forkmann et al., 2014), a mechanism of change that recent research continues to confirm for clients suffering from depression or anxiety disorders (Reinholt et al., 2025). In CBT for social anxiety disorder, changes in cognitive distortions and acceptance of emotions mediated treatment improvement in previous research, highlighting these processes as central targets of therapy (O’Toole et al., 2015). CBT can address maladaptive patterns such as rumination, avoidance, and suppression by encouraging clients to reconsider and reappraise distressing or anxiety-provoking thoughts, thereby reducing the intensity and duration of negative emotional responses. Recent studies have examined emotion regulation strategies as specific mechanisms of change, showing that CBT decreases expressive suppression and increases cognitive reappraisal, which in turn predict reductions in social anxiety (Kivity et al., 2021; Knowles & Tolin, 2024). Other research further extends this perspective by integrating broader emotional constructs—such as emotional intelligence, clarity, and differentiation—into CBT frameworks, suggesting that attention to discrete emotions like shame, embarrassment, and guilt can refine therapeutic targets and improve outcomes (Rozen & Aderka, 2023).

Emotion regulation processes have been recognized as central mechanisms of change in psychotherapy, including both psychodynamic and cognitive-behavioral approaches (Palmieri et al., 2022; Sønderland et al., 2024). For instance, cognitive-behavioral therapy aims to influence clients’ emotion regulation through strategies such as cognitive reappraisal, behavioral activation, and problem-solving (Ryum & Kazantzis, 2024), and research has consistently shown that these interventions can effectively improve clients’ ability to manage emotional experiences (Lancastle et al., 2024). Emotion regulation studies emphasize that such strategies can modulate the intensity, duration, and expression of emotions, providing a framework to understand how therapy facilitates psychological change (Gratz et al., 2015; Savarimuthu et al., 2024).

In addition to emotion regulation processes, individuals hold beliefs about emotions, which refer to their attitudes and assumptions regarding the experience, expression, and management of emotional states (Ford & Gross, 2019; Rozen & Aderka, 2023). Such beliefs can shape whether people attend to, suppress, or express their emotions, and these beliefs are known to influence coping and psychological outcomes (Ceylan & Koc, 2026; Hong & Kangas, 2021). In the context of spiritually integrated therapy, we hypothesize that exploring clients’ beliefs about emotions may reveal how their meaning, spirituality, and religious frameworks interact with emotional experiences. This may provide insight into potential mechanisms through which SPIRIT-CBT facilitates change.

Though there is a need for spiritually integrated therapies and the importance of emotion regulation is well-recognized, the mechanisms and strategies through which spiritually integrated therapies influence emotion regulation remain scarcely understood. It is unclear how the meaning-focused components of these therapies interact with emotion regulation processes, and whether meaning derived from therapy serves as a direct regulatory mechanism for clients’ emotional experiences. Addressing this question is essential to clarify the pathways through which spiritually integrated interventions may impact mental health outcomes.

The following research question was proposed: How does psychotherapy addressing meaning in life, spirituality, and religion (MSR) interact with clients’ emotion regulation?

(a) How does SPIRIT-CBT influence or make clients’ beliefs about expressing, managing, or suppressing emotions related to MSR visible?
(b) How are emotion regulation strategies (e.g., reappraisal, acceptance, distress tolerance) influenced by addressing MSR in therapy?
(c) Does engaging with MSR activate emotion regulation mechanisms for clients’ experienced distress?

Three hypotheses were proposed:

(A) SPIRIT-CBT may provide opportunities for clients to become aware of and to express their emotional experiences in relation to meaning in life, spirituality, and religion (MSR).
(B) SPIRIT-CBT may help to uncover clients’ expressive/behavioral and cognitive emotion regulation strategies in relation to MSR.
  These strategies are derived from established emotion regulation frameworks, particularly within cognitive-behavioral therapy, which emphasize processes such as cognitive reappraisal, acceptance, and distress tolerance, and which are explicitly engaged in SPIRIT-CBT through its cognitive, behavioral, and reflective components.
(C) SPIRIT-CBT may reveal possible emotion regulation mechanisms, including meaning-making, perspective shifting, and narrative processing.
  These mechanisms are theoretically grounded in meaning-oriented and narrative approaches to psychotherapy, which are central to SPIRIT-CBT and explicitly focus on the reinterpretation of experiences, construction of meaning, and integration of life events into coherent narratives.

## 2. Materials and Methods

This study was conducted in the Netherlands as part of a collaborative project between the Center for Research and Innovation in Christian Mental Health Care (Kicg) and four mental health institutions: Altrecht Mental Health Care and GGz Centraal (both regional and secular institutions), and de Hoop and Eleos (both national and Christian institutions). The study adhered to the ethical standards of the Declaration of Helsinki. The medical ethics committee of the University Medical Center Utrecht determined that no further review for Dutch Medical Research Involving Human Subjects was required (NR 22-1041/DB), and the study was approved by the ethics committees of the participating organizations. The project was funded by ZonMw (research number 10960102310035), a national organization for health research and innovation.

### 2.1. Sample and Study Design

A qualitative research design was employed, incorporating two complementary data sources: (1) client evaluation forms (*n* = 118) and (2) in-depth interviews with clients (*n* = 19). All participants were adults with a range of diagnoses, receiving mental health care in acute or clinical settings, and had attended at least one SPIRIT-CBT session (see Table 1). All participants also received other forms of therapy. After participating in the therapy, all respondents were offered evaluation forms, with a response rate of 40–50%, and could indicate whether they consented to be contacted for further research. Either an email address or a phone number was collected, but only in that case. Approximately 20% of clients who completed an evaluation form agreed to be contacted, of whom 19 participated in an in-depth interview. Prior to the interviews, all participants signed informed consent. In the two Christian institutions, a majority of the participants had a Christian identity; in the regular/secular institutions, the outlooks on life were mixed. We obtained information on outlooks on life only from patients in the interviews.

### 2.2. The Intervention

Spiritual psychotherapy for inpatient, residential, and intensive treatment (SPIRIT) was developed as a structured therapeutic group intervention, based on principles of cognitive-behavioral therapy (CBT), and developed for clinical settings, including inpatient, residential, and intensive treatment programs (Rosmarin et al., 2019; C. Marmarosh et al., 2024). A Dutch adaptation of the protocol was used in the current study (van Nieuw Amerongen et al., 2024). Sessions typically lasted 30–55 min, were led by one or two trained clinicians, and could be attended as stand-alone sessions in acute settings or as multi-session programs in residential care. Patients usually did not refuse to participate based on their outlook on life (although R/S affiliation may motivate some in the case of open groups), but the sensitivity of the topics is sometimes perceived as too challenging for them. The primary aim of SPIRIT-CBT was to help clients explore and understand the relationship between meaning in life, spirituality, and religion (MSR) and their mental health. The intervention also supported clients in identifying concrete ways to integrate MSR into their overall treatment plan and in utilizing spiritual or religious concepts to facilitate emotional change. The program acknowledged that MSR could serve as a source of support, but could also be a source of stress or internal struggle, depending on individual context. SPIRIT included both cognitive and behavioral components, supported by handouts. Examples include cognitive restructuring of MSR beliefs, spiritually informed coping strategies, addressing themes such as autonomy and responsibility, prayer, and meditation. Clients were encouraged to actively apply selected concepts or strategies in daily life with the goal of enhancing emotional regulation and coping. Through the combination of meaning-focused and behaviorally oriented interventions, SPIRIT-CBT was explicitly designed to help reduce psychological distress while promoting emotional coherence and well-being, placing the client’s personal spiritual framework at the center of treatment.

### 2.3. Data Collection

Evaluation forms were anonymous and contained five open-ended questions addressing clients’ experiences with the intervention, perceived benefits or struggles, and suggestions for improvement. Semi-structured interview guides covered clients’ experiences with the intervention, perceived impact, barriers and facilitators, and recommendations for further development. Interviews were conducted individually by four different researchers, either face-to-face or online, lasting approximately 45–60 min. The time between the end of therapy and the interviews was approximately two to a maximum of four months. All interviews were conducted after completion of the SPIRIT-CBT treatment and were audio-recorded and transcribed verbatim, with transcripts carefully reviewed and corrected. Based on the interview data, another article was published recently, focusing on impact and aftercare needs (van den Brink et al., 2026). Next to that, a separate analysis will take place regarding patients’ and caregivers’ experiences with SPIRIT-CBT in a broader sense.

### 2.4. Data Analysis

We conducted a thematic content analysis following an iterative and team-based approach (Elo & Kyngäs, 2008; Braun & Clarke, 2024). The analysis proceeded in two complementary coding streams: inductive coding of beliefs about emotions and deductive coding of emotion regulation strategies and mechanisms (as explained below). First, client statements related to their experiences, attitudes, and beliefs regarding emotions were coded without predetermined categories to capture the nuances of how participants perceive and understand emotional experiences. Second, drawing on existing theory and prior research, emotion regulation strategies and underlying regulatory mechanisms were coded using predefined categories (explained below). Strategies were defined as conscious or intentional efforts employed by participants to manage their emotions. The underlying mechanisms were identified as the more abstract processes that support or shape these strategies, and require deeper reflection, facilitated by an in-depth conversation. Emotion regulation strategies were primarily identified through evaluation forms, which provided brief, direct reflections on in-session experiences. Emotion regulation mechanisms, requiring deeper narrative elaboration, were mainly derived from the semi-structured interviews. Although regulatory mechanisms conceptually precede strategies, strategies were coded first because they represent concrete, observable expressions of emotion regulation in the data. This combined inductive–deductive approach allowed us to capture both participants’ lived experiences and the theoretically grounded processes through which SPIRIT-CBT may influence emotion regulation. The first author conducted inductive coding in collaboration with a student research assistant and performed deductive coding with periodic comparisons of interpretations with the second and third authors. Analytic memos documented reflections and emergent insights. Discrepancies were discussed until a shared interpretation of the data was reached.

### 2.5. Theoretical Framework

We distinguished strategies as well as mechanisms. Strategies are coded in five main categories, using a deductive framework derived from established models of emotion regulation:

- Expressive and behavioral strategies: Emotional expression and problem-solving action. Based on coping theory and emotion regulation models, and recent studies that describe emotional expression (Gross, 1998; Zhao & Wang, 2024) and problem-focused action (Cho & Choi, 2024; Lazarus & Folkman, 1985) as behavioral forms of regulation.
- Cognitive strategies: MSR-based reappraisal; positive refocusing; reflection; and rumination. These strategies draw on cognitive models of emotion regulation and recent studies that differentiate adaptive processes such as cognitive reappraisal, positive refocusing, and reflective self-focused processing from maladaptive repetitive thinking patterns such as rumination (Gross, 1998, 2015; Garnefski & Kraaij, 2006; Hu et al., 2024; Nolen-Hoeksema, 1991; Nolen-Hoeksema et al., 2008; Oliva et al., 2023).
- Social strategies: MSR-based connectedness and seeking support. This category is informed by interpersonal emotion regulation theory and empirical findings, which emphasize co-regulation, social connectedness, and support seeking as key regulatory pathways (Do et al., 2025; Zaki & Williams, 2013).
- Acceptance-based strategies: MSR-based acceptance and MSR-based distress tolerance. These codes reflect acceptance-oriented approaches derived from mindfulness- and acceptance-based models such as Acceptance and Commitment Therapy (Hayes et al., 1999) and distress tolerance (Linehan, 1993), which have been recently shown to be powerful strategies (Segal et al., 2025).
- Avoidance and suppression strategies: Suppression and distraction. This category aligns with research showing that both expressive suppression (Gross, 1998), suppression of unwanted thoughts (Wenzlaff & Wegner, 2000), and avoidance-based distraction (Gross, 1998) constitute response-focused strategies that may impede emotional processing (Wang et al., 2024).

Although some authors distinguish bodily or somatic forms of emotion regulation (Ionescu et al., 2025), the present coding framework is organized around functional regulation processes rather than regulatory modalities. Bodily techniques were therefore not included as a separate a priori category. Based on the theoretical focus of the study and the CBT nature of the intervention, it was anticipated that bodily regulation would primarily function as a supporting mechanism within broader regulatory strategies, rather than emerge as a distinct, self-contained strategy. Cognitive strategies were distinguished from acceptance-based strategies because of their different aims: changing the interpretation of a situation (and thereby the emotion) versus allowing and tolerating the emotional experience. Furthermore, meaning in life was not conceptualized as a separate emotion regulation strategy, as it is theoretically understood to function as an underlying mechanism that operates across multiple regulation strategies.

Codes on emotion regulation mechanisms were based on theoretical work on meaning in life, spirituality as coping, hope theory, and narrative emotion regulation (Park, 2010; Pargament, 2013; Baykal et al., 2026). The mechanism codes included:

- Meaning and sense-making. Meaning in life theories describe the process of reinterpreting or reconstructing life events in ways that restore coherence and purpose (Park, 2010; Steger, 2022).
- Hope and anticipatory orientation. Hope theory conceptualizes hope as a future-oriented regulatory mechanism involving goal pursuit and perceived pathways to desired outcomes (Snyder, 2002; Feldman & Jazaieri, 2024; Richardson, 2023).
- Connectedness or belonging. Spiritual and psychosocial coping theories highlight relational and transcendent connectedness as core mechanisms that buffer stress and foster resilience (Pargament, 1997; Ryff & Singer, 2008; Schnell, 2025).
- Control and self-efficacy. Perceived control and self-efficacy function as regulatory mechanisms by shaping appraisals of coping ability and influencing emotional outcomes (Bandura, 1997; Doménech et al., 2024).
- Perspective shifting. Narrative and cognitive models describe perspective shifting as a mechanism that enables psychological distance and reappraisal of emotional experiences (Kross & Ayduk, 2011; Gu et al., 2025).
- Emotional integration and narrative processing. Narrative emotion regulation research shows that constructing coherent stories about emotional events promotes integration and reduces distress (Conway & Pleydell-Pearce, 2000; Pennebaker & Smyth, 2016; Wiesepape et al., 2025).
- Perceived safety. Several theories frame perceived safety and comfort as foundational affect-regulation mechanisms that downregulate threat responses (Mikulincer & Shaver, 2007; Gilbert, 2024).
- Acceptance and tolerance. Acceptance-based models conceptualize openness and willingness toward internal experiences as mechanisms that reduce experiential avoidance and emotional struggle (Hayes et al., 1999; Macri & Rogge, 2024).

## 3. Results

Participant characteristics of the interviews are presented in Table 2. No demographics were collected from the respondents of the evaluation forms.

Results are divided into (1) beliefs about emotions, (2) change in emotion regulation strategies, and (3) meaning in life as a regulatory mechanism. The codes used are shown in Table 3.

### 3.1. Beliefs About Emotions

The evaluation of the therapy revealed that the therapy itself very often evoked emotions. Many of the participants valued this positively, but some experienced difficulties with it. Some indicated that experiencing difficult emotions during the therapy was constructive:

“Difficult, but also good to talk about”

(Evaluation form E10).

One of the participants explicitly mentioned, as a positive, that the therapy touched many clients:

“It also stirred up a lot; it was buried very deeply for many people, so it touched a lot”

(Evaluation form E11).

In addition, the belief was present that suppression would not be helpful and that honest expression was all right:

“Sometimes you actually have to seek out those emotions. It helps to unlock or release something”

(Evaluation form G20).

Others expressed the belief that expressing emotions can have a healing effect and is beneficial:

“Crying. Crying is healing”

(Evaluation form A33).

Some mentioned difficulties with showing emotions, for example, because they did not feel safe enough or did not know the other clients well enough:

“I became a little emotional, which I found a bit difficult for a moment”

(Evaluation form G7).

Sometimes, the emotions of others were experienced negatively:

“Other people’s emotions sometimes make me feel negative”

(Evaluation form E7).

However, another participant stated the following:

“Sometimes more attention could have been given to someone’s grief”

(Evaluation form A28).

One of the participants explicitly mentioned, as a positive, that everything could be there:

“Space for all participants, thoughts, feelings”

(Evaluation form G16).

### 3.2. Emotion Regulation Strategies

Participants demonstrated the use of various emotion regulation strategies during the SPIRIT-CBT sessions or in direct response to the experiences evoked by the sessions. These strategies were categorized into five groups, as explained in the theoretical framework: (1) behavioral, (2) cognitive, (3) social, (4) acceptance-based, and (5) suppressive strategies.

#### 3.2.1. Behavioral Strategies

Participants showed behavioral strategies to cope with their emotions during the therapy. They showed expression, both verbal and non-verbal:

“Sharing thoughts and feelings.”

(Evaluation form E21).

“A quote […] moved me; I had to cry”

(Evaluation form A33).

“I had difficulty sharing at first. This improved later […] It was hard thinking, but also valuable to express these thoughts in words”

(Evaluation form G22).

And they reported problem-solving actions:

“You can do so much for your loved ones”

(Evaluation form E16).

“Yes, to work towards positive goals”

(Evaluation form E17).

“Much flexibility in seeing what there was a need for”

(Evaluation form G19).

#### 3.2.2. Cognitive Strategies

A substantial number of the participants in the evaluation forms showed that SPIRIT-CBT helped them to reflect on their systems of meaning in life, their MSR views and experiences, and the emotions surrounding these systems, views, and experiences:

“You are stimulated; it gets you thinking”

(Evaluation form E19).

“It makes me think, and I can relate to some of the examples”

(Evaluation form H3).

“It is good to talk about it together and gain insight”

(Evaluation form H14).

Often this also had an educational part, either from the leader or other participants, which was highly appreciated:

“Positive, you learn a lot from others”

(Evaluation form E30).

Sometimes they explicitly showed to have new insights by means of this thematic therapy group, which was labeled as MSR-based cognitive reappraisal:

“I liked hearing how others in the same situation think about meaning… I can learn a lot from that”

(Evaluation form A23).

“I started with ‘I have nothing to do with meaning’ and ended with ‘this is important to me/these are my values.’ I can now look at this in an encouraging way”

(Evaluation form G13).

“It was supportive to hear that others are also struggling with questions about meaning. Furthermore, it gave some insight into how the love of others and myself does or does not contribute to meaning”

(Evaluation form G7).

One of the participants realized that emotions are closely linked to behavior and experiences:

“Yes, learning to really listen without judgment. You could see the struggle behind it. I started thinking more about how things work for me and how they have worked in the past”

(Evaluation form E5).

Additionally, positive refocusing regularly was present:

“The openness and nice conversations in the group about the various topics. SPIRIT is a good tool for this and gives support to talk about feelings”

(Evaluation form G20).

A minority showed that the therapy provoked rumination and negativity, which, for them, emphasized meaninglessness and unanswered questions:

“You don’t get answers to your questions about meaning, which is understandable, but it reinforces your frustrations”

(Evaluation form E28).

“It’s hard to leave with unanswered questions”

(Evaluation form E9).

“Questions about family in handout 7… when reading them, my tension increased”

(Evaluation form A14).

“Topics that were too personal came up”

(Evaluation form A24).

“The tension rose quite high, which made it difficult to focus or stay present”

(Evaluation form G16).

“I wonder how much I can/will/want to share in the group, and I get ruminative thoughts about that”

(Evaluation form G9).

The following client explicitly mentioned difficulty:

“The emotions that come up when you listen to or talk about doubts and questions about the ‘why’ and the future”

(Evaluation form E1).

#### 3.2.3. Social Strategies

Participants in evaluation forms often valued social regulation positively: recognition of others, open conversations, and a safe environment helped create a sense of connectedness:

“I was a bit apprehensive about talking about meaning in life beforehand, but it was nice to hear other people’s stories and find some recognition in them, which makes it feel less lonely”

(Evaluation form E1).

“I enjoyed hearing how others in the same situation as me (admitted to acute psychiatry) think about meaning in life at this moment. I can learn a lot from that. I also liked being able to share something myself”

(Evaluation form A23).

“Open atmosphere, room to say your piece”

(Evaluation form H11).

“Pleasant and quite enjoyable to hear about the experiences of others”

(Evaluation form H10).

Their experiences in the group might, in some cases, encourage them to be more open to others in different situations, such as with their loved ones. Some participants made use of the advice of group leaders to seek support after the SPIRIT-CBT group, as shown by the following participant:

“I discussed it afterwards with NN (caregiver). She looked with me at a website where you can see exactly what beliefs each church holds, and so on”

(Client interview 17).

#### 3.2.4. Acceptance-Based Strategies

Participants regularly showed that they were okay with the emotions evoked during the therapy:

“Some topics were tough, but they triggered emotions that were allowed to be there”

(Evaluation form G20).

“It is difficult to be confronted with yourself like that, but of course this is an important step in processing your problems”

(Evaluation form G5).

One of the participants explicitly evaluated the fact, as a positive, that she did not avoid these difficult topics anymore due to the therapy:

“That I don’t avoid the topics anymore”

(Evaluation form E14).

Distress tolerance also took place during therapy:

“I found it difficult to share at first. This improved over time”

(Evaluation form G22).

“Intense to hear how others struggle… Expressing what I never allowed myself to think”

(Evaluation form E5).

“A little hurdle at first. Not easy to feel your own emotions as they emerge”

(Evaluation form A29).

#### 3.2.5. Suppressive Strategies

Suppressive strategies were difficult to capture in the evaluation forms, as participants who decided not to attend or who left the group did not provide responses. In the evaluation forms, some participants appeared to use distraction, combined with positive refocusing or social strategies. One of the interview participants said that he had no benefits from the therapy, possibly reflecting some form of suppression. However, it may also indicate that discussing such personal topics in a group setting felt too overwhelming or intrusive, pointing to the importance of contextual and interpersonal factors in emotion regulation.

“You talk about topics that are quite sensitive… […] I prefer to keep that to myself; I don’t like talking about it”

(Client interview 18).

“The tension was pretty high, which made it hard to stay focused and present”

(Evaluation form G17).

The second quote illustrates the tendency of avoidance, though the client remained present. Most participants clearly reported that SPIRIT-CBT helped them validate or (re)discover emotion regulation strategies, and the interviews showed that underlying mechanisms were often implicitly addressed as well.

### 3.3. Emotion Regulation Mechanisms

As shown in the evaluation forms, MSR-integrated psychotherapy affects internal emotion regulation strategies by reducing distress, supporting cognitive reappraisal, and enhancing feelings of control and connectedness. Additionally, the interviews revealed that emotion regulation mechanisms were also activated—and at times shifted—during or after the therapy.

Across interviews, the responses of the participants indicated a set of implicit psychological processes that were activated when engaging with the content of the therapy. These processes do not reflect deliberate coping strategies but rather deeper mechanisms of emotion regulation that emerge through the exploration of meaning in life, spirituality, and religiosity. Eight processes were identified.

First, the experience of a sense of meaning in life was strengthened as participants tried to situate their suffering within a broader existential or spiritual narrative, which appeared to reduce emotional chaos and promote coherence:

“Within the SPIRIT-group I got the feeling that that was basically okay too… small things give life meaning”

(Client interview 14).

Second, perspective shifting occurred as the dialogues helped participants view their experiences from a more compassionate or transcendent vantage point, creating cognitive distance from distress:

“So through the therapy I have learned to see differently, a way of looking that things can simply be very different.” And “That session and that remark from the other person stimulated my reflections. And it made me start thinking from a different perspective”

(Client interview 17).

Third, acceptance and tolerance of painful internal states increased, often triggered by discussions of religious or spiritual struggles, suffering, or questions about meaning in life. This acceptance and tolerance helped clients experience their emotions without escalation:

“Yes, absolutely, yes, absolutely, it has changed me… It has changed me in the sense that feelings exist, and that’s okay. But it’s about how you deal with them. […] That it was simply acknowledged, and that now I can even say to myself, I am allowed to be angry—and I can still exist before You? […] That you can come to God with your questions, and you don’t immediately need a yes or no answer. That it’s okay to discuss it, so to speak. That you are allowed to discuss your faith, your life of faith, your questions about faith”

(Client interview 6).

This quote illustrates that connection is also about acceptance and being in relation to others.

Fourth, participants described enhanced connectedness, both to others in the group and to something larger than themselves, fostering felt support and decreasing isolation:

“I think that, for me—and I don’t know if that was really the intention of the SPIRIT group—but for me, it was kind of like this. I’m trying to put it into better words: when it came to loneliness, I realized I wasn’t alone in it, and I really appreciated being able to talk about it in the group. The loneliness I felt, the fact that it was shared, that so many people experienced it too… even the group leaders shared that as well. Loneliness can appear in so many different ways. Even if you have a good social network, you can still feel lonely. For me, it also lifted a huge weight that I could express my pain and my struggle with faith. I had never been able to share that like this before—and here, I could”

(Client interview 14).

The fact that group leaders sometimes showed their personal experiences seemed to strengthen the experience of connectedness.

Fifth, many reported an emergent sense of comfort or safety, which seemed grounded in perceiving transcendent presence, care, or guidance—reducing fear and emotional reactivity:

“For me, it is the comfort it provides and also the sense of future. Faith plays a big role in my daily life. And the SPIRIT-group also helps me to engage more with that aspect of faith—asking questions like, ‘What is the path that God has planned for me? […] For a long time, I felt support. However, in the past few weeks, I felt more struggle. But I notice that after the last SPIRIT-group session, it is starting to shift back towards support again. […] That’s more in the feeling, I think. That I’m engaging with it, and that I actually feel comforted by faith—and that it gives me hope”

(Client interview 3).

Sixth, narrative integration unfolded as participants wove difficult life events into a more coherent life story, reducing fragmentation and offering a sense of continuity:

“I started reflecting a lot on the past. Those insights […] came through the song and the conversation. […] I already knew that, but I began to really feel it after that group. […] The conversation I had […] really opened my eyes to not only talk about the present but also about the past—sharing more, so to speak”

(Client interview 13).

Seventh, moments of hope appeared when participants reframed their situation in light of future possibilities, spiritual trust, or signs of meaning in life, providing motivational energy in moments of despair:

“It has definitely influenced my illness. Sometimes you see things on paper that resonate […] And those really were there. In the handouts, but also often in what other clients mentioned. Sometimes unexpected perspectives came up that really made you think […] And that doesn’t happen, or happens less, in other therapies. This was quite intimate. […] At the beginning, when things were really bad, I was really in my head thinking: what am I supposed to learn from this situation? Am I being punished? Or… what am I supposed to learn on a soul level from this? Haven’t I gone through enough problems already? Will it ever stop? And I knew that too, but then here at the SPIRIT-group, you were reinforced to think: yes, nothing stays the same. That’s one of the things I’ve learned. Nothing stays as it is. Everything is subject to change—in nature, in dealing with people, and in dealing with myself. What you feel today doesn’t mean that in a month or two I’ll feel the same. And I can see that now. I mean, yes. Back then I could only bike for half a minute, and now I biked here’’

(Client interview 2).

“… that gives me hope. That God has things in His hands, that He has already shaped my path, and that things like depression and suicidal thoughts are ultimately not part of that and are, in principle, finite”

(Client interview 3).

Finally, self-efficacy increased implicitly, as engaging with difficult existential themes reduced avoidance and fostered a sense of agency in navigating emotional or spiritual challenges:

“The whole process certainly helped me. But not necessarily the meaning-making aspect. It’s mostly about how I deal with my feelings and how I notice things happening in my body—so when an uneasy feeling arises, I don’t immediately think, ‘Oh, I have to do something else now.’ I let myself experience it. And that’s primarily about thinking in a healthy, calm way. That has helped me a lot”

(Client interview 8).

Although the participant does not explicitly attribute this learning to the meaning in life component of SPIRIT-CBT, the structured group setting and guided reflection may have indirectly supported the development of self-efficacy in managing emotions and bodily sensations. The participant emphasized a sense of calm and the ability to let emotions arise without immediate reaction. This suggests that beyond active meaning-making, the therapy may facilitate contemplative processes that support emotion regulation.

Some general findings also emerged from the analysis. The type of institution (implicating the setting of the group and group leader) mattered in the extent to which and the way emotion regulation strategies were touched. In addition, the evaluation forms showed that taking the handouts home can evoke emotional responses that are not processed in the group.

## 4. Discussion

The findings of this study indicate that psychotherapy addressing meaning in life, spirituality, and religion (MSR), as provided by SPIRIT-CBT, interacts with emotion regulation at different levels. Overall, the results largely confirmed the hypotheses, suggesting that addressing MSR in therapy (1) has the potential to increase awareness of emotions and makes implicit beliefs about emotions explicit, and (2) engages emotion regulation strategies and (3) underlying mechanisms. One could argue that beliefs and thoughts about emotions constitute the deepest level, shaping both regulation mechanisms and regulation strategies (Ceylan & Koc, 2026; Ford & Gross, 2019; Hong & Kangas, 2021; Koc & Uzun, 2024).

With regard to the first research question, the findings supported the hypothesis that SPIRIT-CBT increases awareness of MSR-related emotions and provides space to express them. The emotions that emerged, such as sadness, fear, anger, and hope, might not be experienced as fundamentally different from emotions arising in other life domains, but their meaning, object, and existential scope were shaped by their framework of MSR. For many individuals, MSR is related to the sacred, which is in some way different from other human experiences that are also meaningful and valuable because it relates to mystery, ineffability, transcendence, deep interconnectedness, and profound purpose in our lives (Pargament & Pomerleau, 2025). Previous research has shown that MSR struggles can be strongly associated with emotional distress (van Nieuw Amerongen-Meeuse et al., 2020). For example, fear may involve fear of evil, guilt may take the form of moral guilt, and hope may extend beyond the boundaries of life itself (Exline et al., 2000; Wilt et al., 2022). The present findings suggest that SPIRIT-CBT created space to articulate and reflect on these issues rather than leaving them implicit or unexamined. Previous research has shown that beliefs about emotions are strongly related to emotion regulation strategies (Hong & Kangas, 2021; Koc & Uzun, 2024).

Regarding the second research question, results indicated that participants employed the hypothesized cognitive and expressive/behavioral emotion regulation strategies, while social, acceptance, and avoidance strategies were also observed. Participants showed various examples of cognitive reappraisal, which may be due to the group context, which allowed the sharing of multiple perspectives. This might be beneficial for treatment outcomes, while high cognitive reappraisal is associated with stronger improvements in quality of life (Barrio-Martínez et al., 2022). Sharing is not always related to better health outcomes (Pauw et al., 2025), but cognitive reappraisal strategies in an earlier study have been reported to be more effective for regulating emotions than, for example, suppression (Webb et al., 2012). More recent evidence from interpersonal emotion regulation research indicates that both reappraisal and suppression can reduce negative emotions in others, highlighting contextual variation in strategy effectiveness (Wang & Shi, 2025). Acceptance-based emotion regulation strategies have been associated with reductions in anxiety and psychological distress (Zhang et al., 2023). Many participants validated the experienced connection, including with the group leaders. It is possible that group leaders who facilitate SPIRIT-CBT groups were also more comfortable sharing personal insights, which contributed to this sense of connection. The strategies observed in the current study, like the emotions themselves, did not constitute a separate class of MSR-specific regulation strategies, but rather reflected common regulatory processes grounded in a transcendent or sacred source of meaning in life (Pargament, 2013; Pargament & Pomerleau, 2025). The key differences lie in the source or authority (external rather than internal, transcendent rather than intrapsychic), the MSR-related content, and the extended time horizon, which may reach into eternity (Vishkin, 2021). For instance, reappraisal may take the form of “God has a plan,” acceptance may involve the belief that “this suffering is allowed and has a purpose,” and social regulation may be directed toward a religious community.

In the interviews, it became apparent that these strategies were underpinned by more fundamental mechanisms, which confirmed the hypothesis of the third research question. It was at the level of regulation mechanisms that MSR became truly distinctive. MSR influenced meta-beliefs about emotions, existential safety, moral frameworks, and the experienced tension between temporality and transcendence. This is congruent with Pargament and Pomerleau (2025), who conceptualize religion and spirituality as powerful, dynamic meaning systems centered on the sacred, which can help individuals navigate life’s challenges. Following the PROCESS emotion regulation model of Gross (2015), which is still used for research and practice (Bachfischer & Harris, 2025), the different aspects of emotion regulation as being induced by SPIRIT-CBT could potentially take place in various phases. During the identification stage, clients might become aware of certain emotions and find room to express them or their beliefs about them (e.g., guilt, existential anxiety, or hope). During the implementation stage of SPIRIT-CBT, the group setting might help to find a safe space to share emotions and apply (new) regulation strategies, such as perspective shifting. The monitoring phase could be seen as the stage of narrative processing and a reflection of former and current MSR values.

Pargament and Pomerleau (2025), however, also clearly noted that MSR may become a source of struggle when meaning in life is threatened. The clinical relevance of religious/spiritual (R/S) struggles emphasizes the relevance of paying attention to MSR in therapy, including R/S struggles. R/S struggles have been associated with increased psychological distress and various forms of psychopathology, including symptoms of depression and anxiety (Wilt et al., 2022). Such struggles may challenge individuals’ core meaning systems and beliefs, potentially intensifying negative emotional responses to stressful life events. From a psychological perspective, these processes may partly operate through emotion regulation mechanisms, as spiritually threatening interpretations—such as perceiving divine punishment—can reinforce maladaptive regulation strategies like rumination or avoidance (Exline et al., 2014; Brandão, 2025). Beyond its aim to address the unaddressed and to draw on sources of hope, safety, and comfort, spiritually integrated approaches such as SPIRIT-CBT also aim to work with the dynamics of R/S struggles. This may be achieved by facilitating meaning-making and fostering more adaptive reinterpretations of MSR experiences, thereby supporting helpful emotion regulation and strengthening overall psychological functioning.

Our findings align with the literature on positive emotions and the role of MSR. Engagement with MSR themes has been shown to elicit positive emotional experiences and support adaptive emotion regulation strategies in therapy (Van Cappellen et al., 2023; Lee et al., 2022). Although a review of previous quantitative studies reports only small-to-moderate associations between MSR and emotion regulation (Brandão, 2025), our findings suggest that MSR themes are indeed potentially relevant for emotional processes. Emotion regulation has also been linked to self-direction and meaning in life within personality theory, a relationship that has been elaborated in detail by Steen et al. (2024). This suggests potential parallels between processes of self-regulation, personality development, and meaning in life. However, the domain of worldview, meaning in life, and purpose is difficult to capture quantitatively. Our qualitative results show that clients in SPIRIT-CBT actively engage with these processes and develop new regulation strategies, indicating a richer and more dynamic interplay than could be observed in questionnaire-based research.

What makes SPIRIT-CBT relevant is that it provides space for themes that are often overlooked in standard care, but which are closely linked to many clients’ complaints (Exline et al., 2000, 2014; Perry, 2024) and that there is a need to address (van Nieuw Amerongen-Meeuse et al., 2020; Gazaway et al., 2026). By making these previously unaddressed topics discussable, clients are given the opportunity to reframe their experiences. They may learn to accept certain feelings (which can be facilitated by recognizing them in others) and become less able to avoid these issues. Consistent with body-based and trauma-informed perspectives (van der Kolk, 2014; C. L. Marmarosh et al., 2022), the therapeutic impact of the group may lie in the shared attunement created through openly allowing and reflecting on emotions and thoughts, fostering co-regulation, safety, and meaning in life within a spiritually informed context. At the same time, group therapy is not suitable for everyone, and challenges such as emotional tension and suppression may persist. It is therefore essential to carefully tailor care to the individual needs of each client.

### 4.1. Clinical Implications

The current study supports the clinical relevance of integrating MSR-focused psychotherapies within existing treatment settings. The three areas of the current study may guide clinicians in addressing MSR. When basic emotion regulation mechanisms are healthy, they may be reactivated and restored after periods of MSR-related distress. However, when these mechanisms are not well developed, progress may take longer, requiring a stepwise approach: encouraging emotional expression, reviewing and practicing regulation strategies, and fostering healthy mechanisms over time. Therefore, it is important that therapists providing SPIRIT-CBT or addressing MSR individually pay attention to clients’ MSR-related beliefs about emotions—for example, whether experienced anger, anxiety, or doubt is perceived as acceptable or immoral. The SPIRIT-CBT group setting offers the opportunity to explore diverse perspectives, both from the group and from external sources, in a non-directive way. The initial step of identification and acceptance of certain emotions may help clients consider ways to manage these emotions in a manner compatible with their MSR framework. In addition, clinicians may realize that MSR struggles may be deep (e.g., in cases of trauma) and cannot always be addressed during (group) therapy. For some, topics may be too painful and a therapeutic approach may not suit in all cases. It is therefore recommended that clinicians, whenever possible, take an MSR-focused history with individual clients to map this. Consultation of spiritual caregivers should easily be considered (Akwa GGz, 2023). SPIRIT-CBT is essentially designed as a single-session group format, with the possibility for clients to participate in multiple consecutive groups, often without prior individual contact with group leaders. This highlights the importance of follow-up to assess the impact of the therapy and to support clients in consolidating these effects, potentially stepping up to individual therapy if needed (van den Brink et al., 2026).

### 4.2. Limitations and Future Directions

The present study was designed to evaluate SPIRIT-CBT and not specifically to investigate emotion regulation. This was both a strength and a potential limitation: the analysis could only be based on what spontaneously emerged in the data regarding these topics (Patton, 2015; Polat, 2025). Participants were drawn from four different clinics, in treatment settings varying from day care to acute care and longer stay clinical care, where the therapy was delivered by group leaders from various disciplines. This introduced a high degree of contextual variation, resulting in a broad and overarching picture rather than an in-depth analysis within a homogeneous client population (Patton, 2015; Polat, 2025). At the same time, this aligned with the flexible and widely applicable approach of the protocol. In this study, strategies were examined within the therapy context, not outside the group setting. If the study had been specifically designed to examine emotion regulation, additional findings might have emerged. Regulation mechanisms may also require more time to develop or become observable, if measurable (Voelkle et al., 2018; MacNamara et al., 2023). The participants’ characteristics showed that clients with mood disorders might be more represented than others, raising the question of whether different diagnostic groups, such as clients with personality disorders, might profit from other approaches than CBT (Setkowski et al., 2023). Finally, participants were likely those who derived benefit from the therapy. Individuals who continue to avoid or suppress emotions were likely to be underrepresented, and these strategies may limit engagement in and benefit from group-based support within SPIRIT-CBT (Wang et al., 2024). The current study lacks data on those who did not engage with, or who dropped out of, the therapy.

For future studies, it could be valuable to conduct a questionnaire-based study measuring the effects of therapy addressing MSR themes on religious struggles, given their known close association with emotion regulation (van Nieuw Amerongen et al., 2024). Longitudinal follow-up of individuals could also help clarify how changes in emotion regulation strategies and mechanisms unfold, and test whether the perspective on beliefs, strategies, and underlying mechanisms is accurate. Future research could explore which individuals experience MSR-related emotion regulation difficulties, the types of emotions involved, and which interventions are most effective in alleviating these difficulties for various diagnostic groups (Berking & Wupperman, 2012; MacNamara et al., 2023).

## 5. Conclusions

Overall, the results indicate that attention to MSR themes heightens clients’ awareness of their relevance for mental health and engages a broad range of emotion regulation strategies, as well as possibly underlying mechanisms. These results partially support our hypotheses. MSR-related well-being seems to be associated with emotion regulation strategies and mechanisms. As hypothesized, SPIRIT-CBT brought MSR into focus, allowing participants to articulate and reflect on beliefs about emotions and to practice regulation strategies in a safe group environment. These findings support hypothesis A. Participants reported using a range of strategies, including social and cognitive strategies, but also acceptance of painful states and behavioral expression. These findings provide partial support for hypothesis B. Engagement with MSR also appeared to be related to deeper mechanisms. Indeed, as expected, meaning-making, perspective shifting, and narrative processing were present, but also enhanced tolerance of distress, increased connectedness, and a sense of safety or comfort grounded in belief and values. These findings support hypothesis C.

Taken together, these findings suggest that integrating MSR themes into psychotherapy exerts effects beyond cognitive or reflective processes and intertwines with emotion regulation processes that shape how distress is experienced, tolerated, and potentially transformed.

## Figures and Tables

**Table 1:** Dataset and sample.

	Altrecht	GGz Centraal	De Hoop	Eleos
Identity of clinic	Regular	Regular	Christian	Christian
Group setting	Clinical stay	Day care	Day care	Clinical stay
Frequency	Biweekly	Weekly	Weekly	Weekly/biweekly
Sessions	Open	Open	Fixed (10 sessions)	Fixed (2 sessions)
Evaluation forms (N)	33	26	25	34
Interviews	9	4	3	3

**Table 2:** Participant characteristics from the interviews.

	Clients (N = 19)
**Sex (n)**	
Male	8
Female	10
Nonbinary	1
**Age, mean (SD)**	41 (14)
**Context group (n)**	
Clinical unit	16
Day care	3
**Outlook on life (n)**	
Christian, orthodox	4
Christian, evangelical	2
Islamic	1
Nonreligious	12
Diagnoses (n self-reported) *	
Depression	11
PTSD	8
Substance use	3
Psychotic disorder	3
Anorexia	2
Anxiety disorder	2
Autism	2
ADHD	1
Bipolar disorder	1

* Totals exceed N because participants could report multiple diagnoses.

**Table 3:** Overview of inductive and deductive codes used in the analysis.

Core Code	Subcode	
	Sharing of emotions is useful	
	Sharing is heavy but beneficial	
	Suppression of emotions is not helpful	
**Beliefs (inductive)**	Honest expression of emotions is all right	
	Sharing is not safe/too difficult	
	Paying attention to emotions is important	
	Showing emotions gives discomfort	
	Others’ emotions negatively affect me	
**Core Code**	**Grouping Code**	**Subcode**
**Regulation strategies (deductive)**	Expressive/behavioral strategies	Emotional expression
		Problem-solving action
	Cognitive strategies	Reflection
		Positive refocusing
		MSR-based reappraisal
		Rumination
	Social/interpersonal strategies	MSR-based connectedness
		Seeking support
	Acceptance-based strategies	MSR-based acceptance
		MSR-based distress tolerance
	Avoidance/suppression strategies	Suppression
		Distraction
		Avoidance
**Core Code**	**Subcode**	
**Regulatory mechanisms (deductive)**	Meaning in life and sense-making	
	Hope and anticipatory mechanism	
	Connectedness and social belonging	
	Control and self-efficacy	
	Perspective shifting and cognitive distancing	
	Emotional integration and narrative processing	
	Comfort and safety	
	Acceptance and tolerance	

## Data Availability

Data is anonymized and stored at the Center for Research and Innovation of Christian Mental Health Care and is available at reasonable request.

## Abbreviations

The following abbreviations are used in this manuscript:

SPIRIT	Spiritual psychotherapy for inpatient, residential and intensive treatment
CBT	Cognitive-behavioral therapy
MSR	Meaning in life, spirituality and religiosity
R/S	Religious/spiritual
